# Synthesis and crystal structure study of (*R*,*R*)-TMCDA ethanol derivatives doubly protonated with FeCl_4_^−^ and Cl^−^ as counter-ions

**DOI:** 10.1107/S2056989025003019

**Published:** 2025-04-08

**Authors:** Franziska Dorothea Klotz, Clara Alonso Felipe, Fernando Villafañe, Carsten Strohmann

**Affiliations:** a Inorganic Chemistry, TU Dortmund University, Otto-Hahn Str. 6, 44227 Dortmund, Germany; b Química Inorgánica, Universidad de Valladolid, Paseo Belén 7, 47011 Valladolid, Spain; Venezuelan Institute of Scientific Research, Venezuela

**Keywords:** crystal structure, (*R*,*R*)-TMCDA, stereogenic carbon center, hydrogen bonds, (*R*,*R*)-TMCDA ethanol derivative

## Abstract

The synthesis and structural characterization of the crystal forms of (*R*,*R*)-TMCDA and its ethanol derivative, both doubly protonated with FeCl_4_^−^ and Cl^−^ as counter-ions, are reported, namely, (*R*,*R*)-*N*^1^,*N*^1^,*N*^2^,*N*^2^-tetra­methyl­cyclo­hexane-1,2-bis­(aminium) tetra­chlorido­ferrate chloride, (C_10_H_24_N_2_)[FeCl_4_]Cl (**1a**), and (*R*,*R*)-*N*^1^-(2-hy­droxy­eth­yl)-*N*^1^,*N*^2^,*N*^2^-tri­methyl­cyclo­hexane-1,2-bis­(aminium) tetra­chlorido­ferrate chloride (C_11_H_26_N_2_O)[FeCl_4_]Cl (**2a**). A notable feature across both synthesized compounds is the presence of N—H⋯Cl hydrogen bonds of moderate strength in the solid state. In the case of the ethanol derivative of (*R*,*R*)-TMCDA, the structure also reveals the formation of inter­molecular O—H⋯Cl hydrogen bonds.

## Abstract 2

The synthesis and structural characterization of the crystal forms of (*R*,*R*)-TMCDA and its ethanol derivative, both doubly protonated with FeCl_4_^−^ and Cl^−^ as counter-ions, are reported. A notable feature across both synthesized compounds is the presence of N—H⋯Cl hydrogen bonds of moderate strength in the solid state. In the case of the ethanol derivative of (*R*,*R*)-TMCDA, the structure also reveals the formation of inter­molecular O–H⋯Cl hydrogen bonds.

## 1. Chemical context

Nitro­gen-containing compounds have numerous applications in coordination chemistry. Of particular inter­est is the mol­ecule (*R*,*R*)-TMCDA (**1**), which contains two stereogenic carbon centers, enabling it to function as a bidentate, chiral ligand.

Selective deprotonation reactions play a critical role in the functionalization of certain mol­ecules, facilitating the incorporation of new functional groups and enhancing mol­ecular properties.

In organolithium chemistry, polyamines are the only tertiary amines susceptible to deprotonation reactions. This is due to the fact that polyamines allow the formation of a complex in which the li­thia­ted base gets pre-coordinated. This pre-coordination brings reactive groups into close proximity, allowing selective deprotonation reactions to take place (Gessner *et al.*, 2010 ▸). This phenomenon is widely known as the complex-induced proximity effect (CIPE) (Gessner *et al.*, 2010 ▸; Whisler *et al.*, 2004 ▸; Breit, 2000 ▸; Hoveyda *et al.*, 1993 ▸; Beak & Meyers, 1986 ▸).

Additionally, the synthesis of new ligands with stereogenic centers presents a persistent challenge. The inherent chirality of (*R*,*R*)-TMCDA (**1**) coupled with its high reactivity towards li­thia­ted bases, renders this mol­ecule a promising precursor for the synthesis of other novel ligands. A notable example is compound **2**, which displays inter­esting characteristics including chirality and its function as an *N*,*N*,*O*-scorpionate ligand. In light of these characteristics, our research group has previously investigated compound **2** and related ligands (Gessner *et al.*, 2010 ▸) as well as with (*R*,*R*)-TMCDA (Eckert *et al.*, 2011 ▸; Strohmann & Gessner, 2008 ▸; Strohmann & Gessner, 2007 ▸). Building upon this foundation, compounds **1a** and **2a**, incorporating the aforementioned ligands, have been successfully synthesized and crystallized in this work.
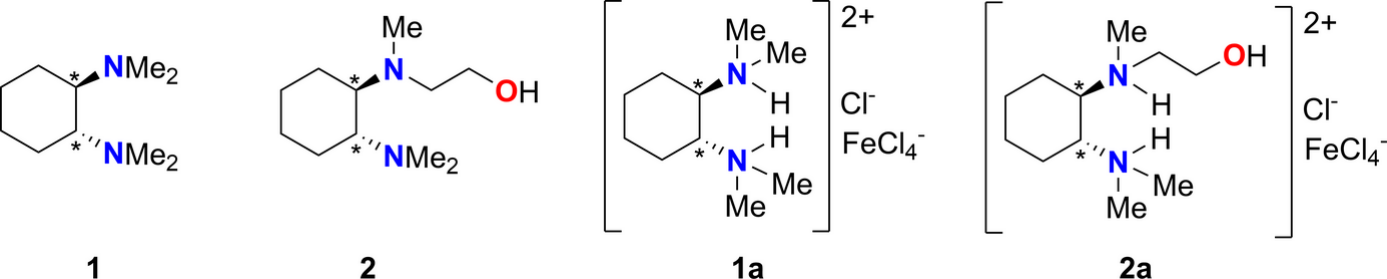


## 2. Structural commentary

**Figure 1:**
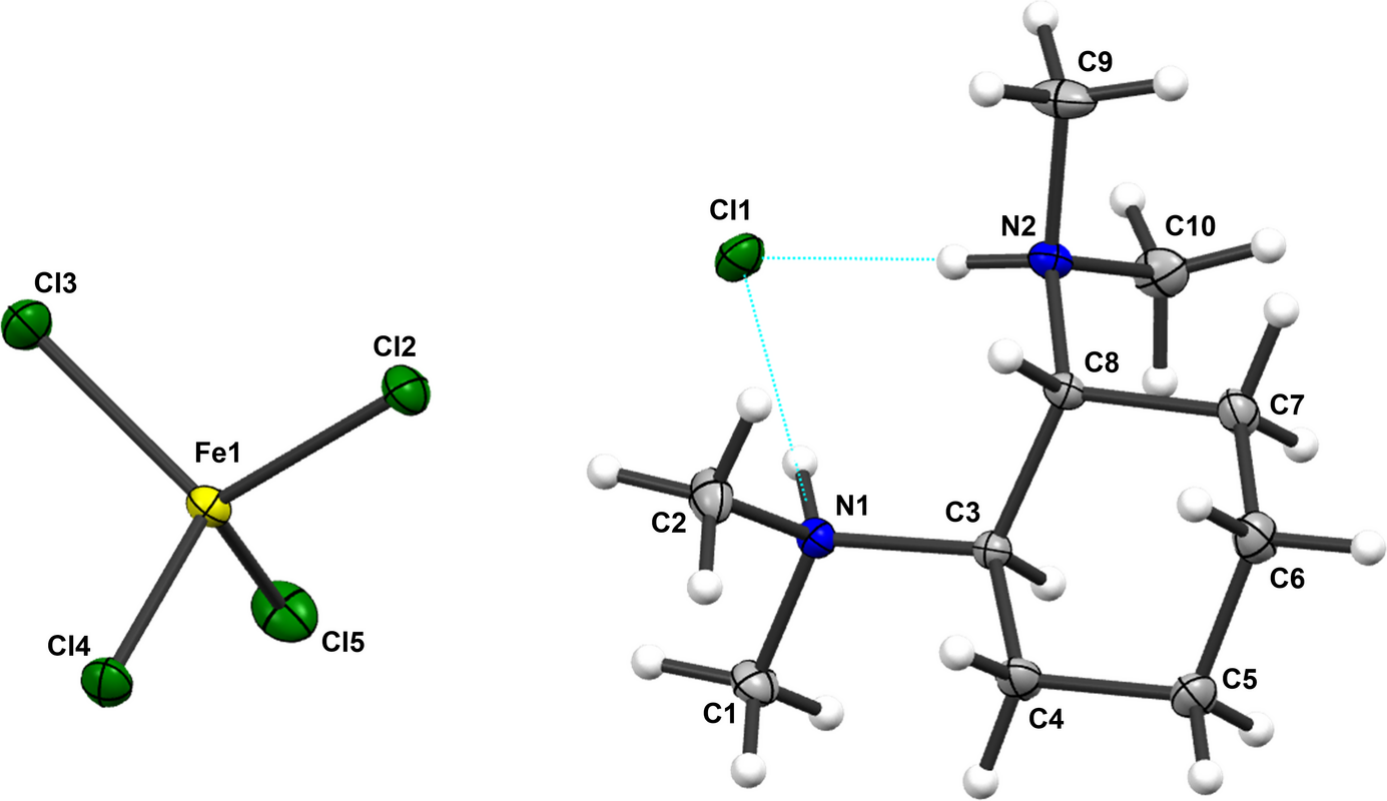
The mol­ecular structure of compound **1a** with the atom labelling and displacement ellipsoids drawn at the 50% probability level.

The structure of compound **1a**, as depicted in Fig. 1 ▸, comprises a cation and two anions. The cation consists of a doubly protonated (*R*,*R*)-TMCDA mol­ecule, with the cyclo­hexane ring adopting a chair conformation. The two counter-ions are a chloride anion, that forms two N—H⋯Cl hydrogen bonds, and a FeCl_4_^−^ anion, in which the Fe^3+^ cation is coordinated by four chlorides in a tetra­hedral geometry.

The distances of inter­est are shown in Table 1 ▸. Hydrogen bonds between the chloride and the hydrogen atoms bonded to either N1 or N2 were observed in the crystal structure, with N1—H1⋯Cl1 distances of 2.133 (19) Å and N2—H2⋯Cl1 distances of 2.135 (17) Å and N1—H1 distances of 0.891 (19) Å and N2—H2 distances of 0.900 (18) Å. These hydrogen bonds could be classified as moderate (Steiner, 2002 ▸).

**Figure 2:**
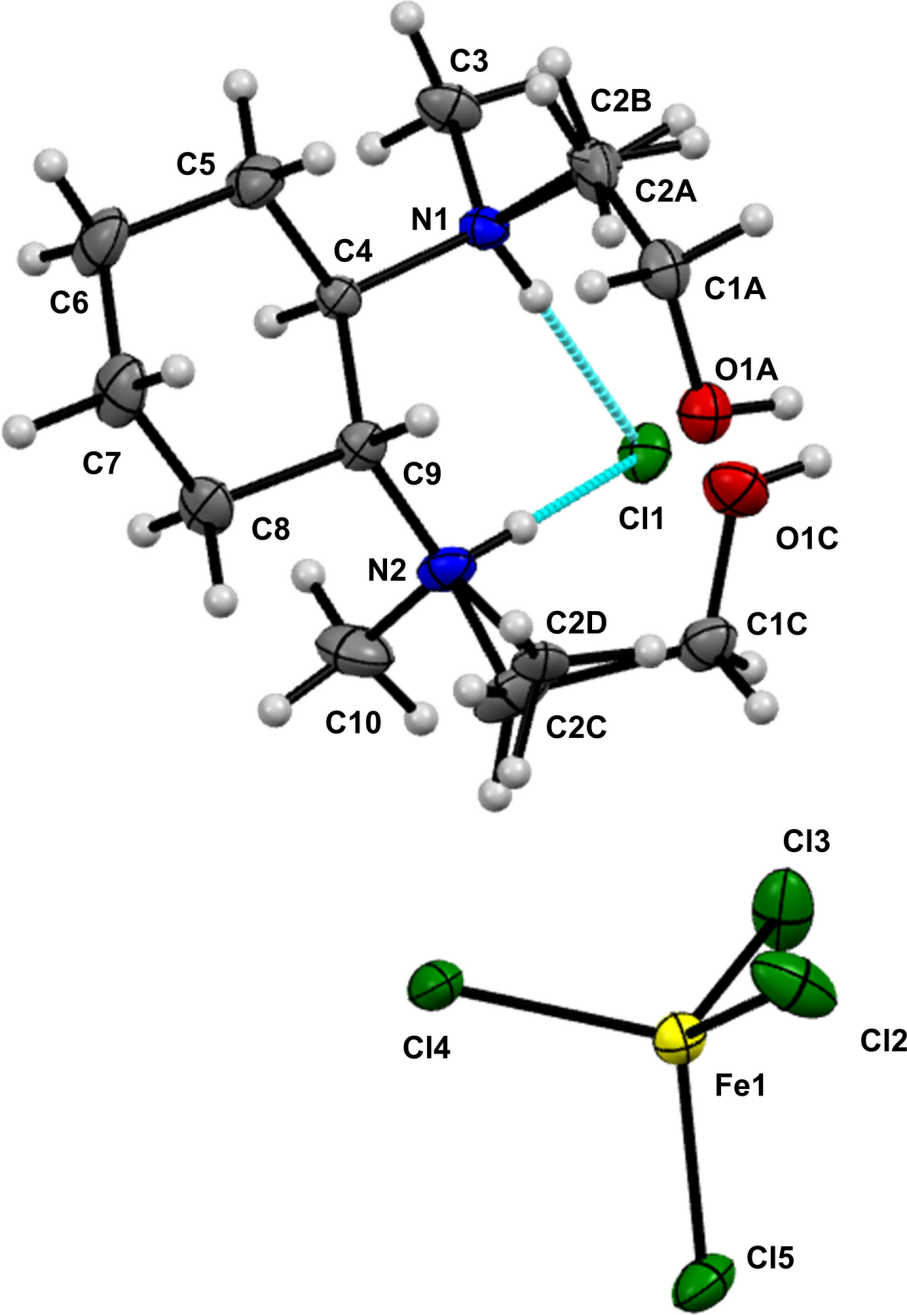
The mol­ecular structure of compound **2a** with the atom labelling. The structure exhibits a disorder of the –CH_2_CH_2_OH fragment at two positions, with occupancies of 0.4 and 0.6 and a disorder of C2, also at two positions with occupancies of 0.4 and 0.6.

The structure of compound **2a**, illustrated in Fig. 2 ▸, consists of a cation and two anions. The cation is derived from an ethanol-substituted, doubly protonated (*R*,*R*)-TMCDA mol­ecule, characterized by a cyclo­hexane ring adopting a chair conformation. The two counter-ions include a chloride, which forms N—H⋯Cl hydrogen bonds, and a FeCl_4_^−^ anion, wherein the Fe^3+^ cation is coordinated by four chlorides arranged in a tetra­hedral geometry. The cation in this structure features three tetra­hedral stereogenic centres: the two carbon atoms associated with the N-bonded cycle and the nitro­gen atoms N1 or N2, which are bonded to the ethanol fragment. The nitro­gen atoms have a labile configuration and both enanti­omers (*R*_N_ and *S*_N_) can occur in solid state and thus represent a further cause of the existing disorder. The more flexible CH_2_-segments of the side chain contribute to the observed disorder of the –CH_2_CH_2_OH fragment as well. This is distributed at atom C2 over positions designated as *A* and *C* (CH_2_ group) as well as *B* and *D* (CH_3_ group) with occupancies of 0.6 and 0.4 respectively. Additionally, the C1 carbon is similarly disordered at positions *A* and *C*, also exhibiting occupancies of 0.6 and 0.4 respectively. The relevant distances are summarized in Table 2 ▸. The structural analysis reveals hydrogen bonds between the chloride anion and the hydrogen atoms attached to nitro­gen atoms N1 and N2. The N2—H2⋯Cl1 distance is measured at 2.357 (3) Å, while the N1—H1⋯Cl1 distance is 2.221 (3) Å. Additionally, the N1—H1 distance is 0.855 (3) Å, and the N2—H2 distance is 0.878 (3) Å. These hydrogen bonds can be classified as moderate in strength, according to Steiner (Steiner, 2002 ▸). In contrast, no inter­action is observed between the hydrogen atoms O1*A*—H1*A* or O1*C*—H1*C* and Cl1, as they are separated by a distance of 3.1245 (5) Å (H1*A*⋯Cl1) and 3.4958 (5) Å (H1*C*⋯Cl1).

## 3. Supra­molecular features

**Figure 3:**
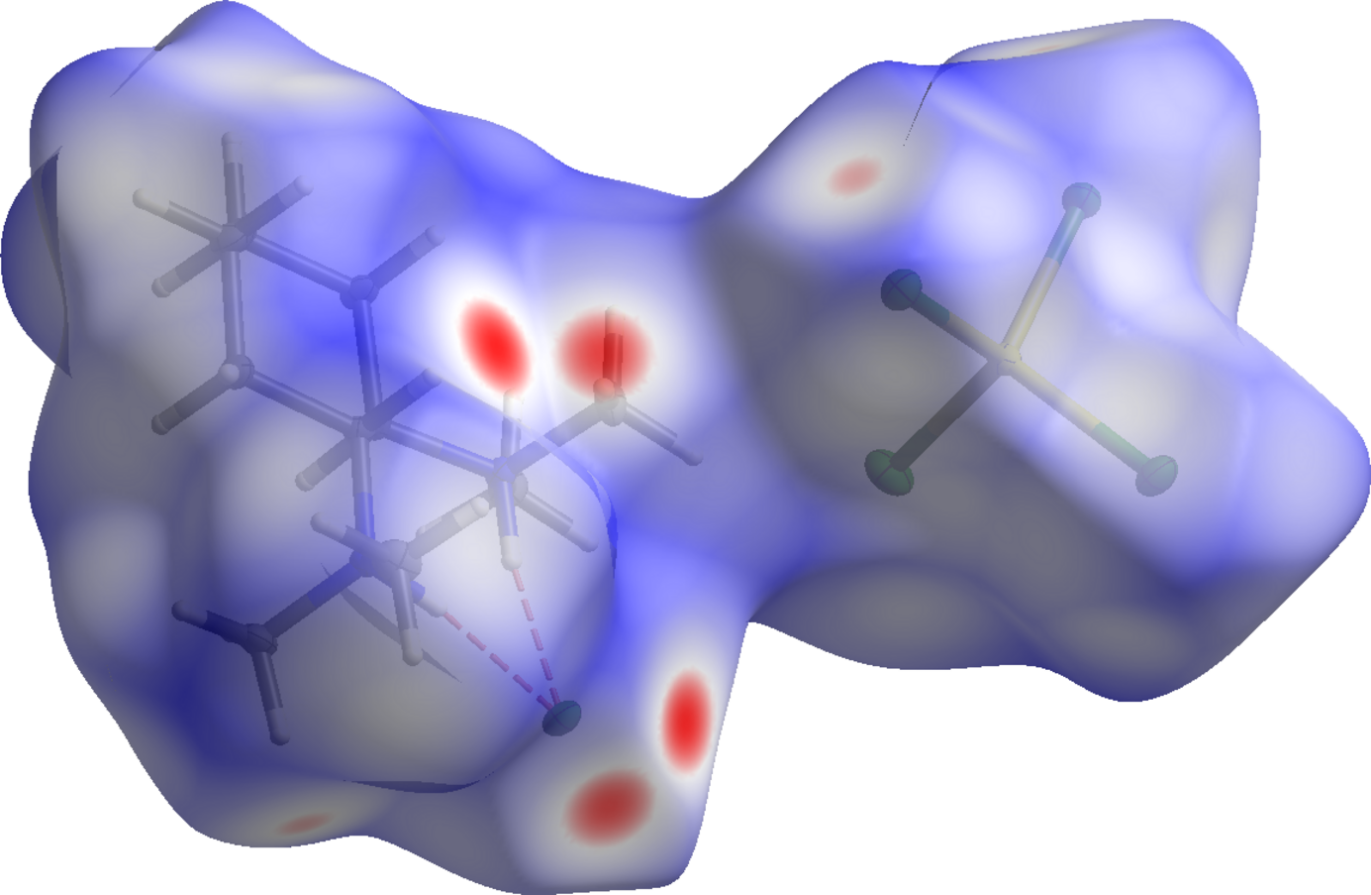
Three-dimensional Hirshfeld surface of **1a** mapped over *d*_norm_ (rescale surface property: −0.2124 − 1.4372).

**Figure 4:**
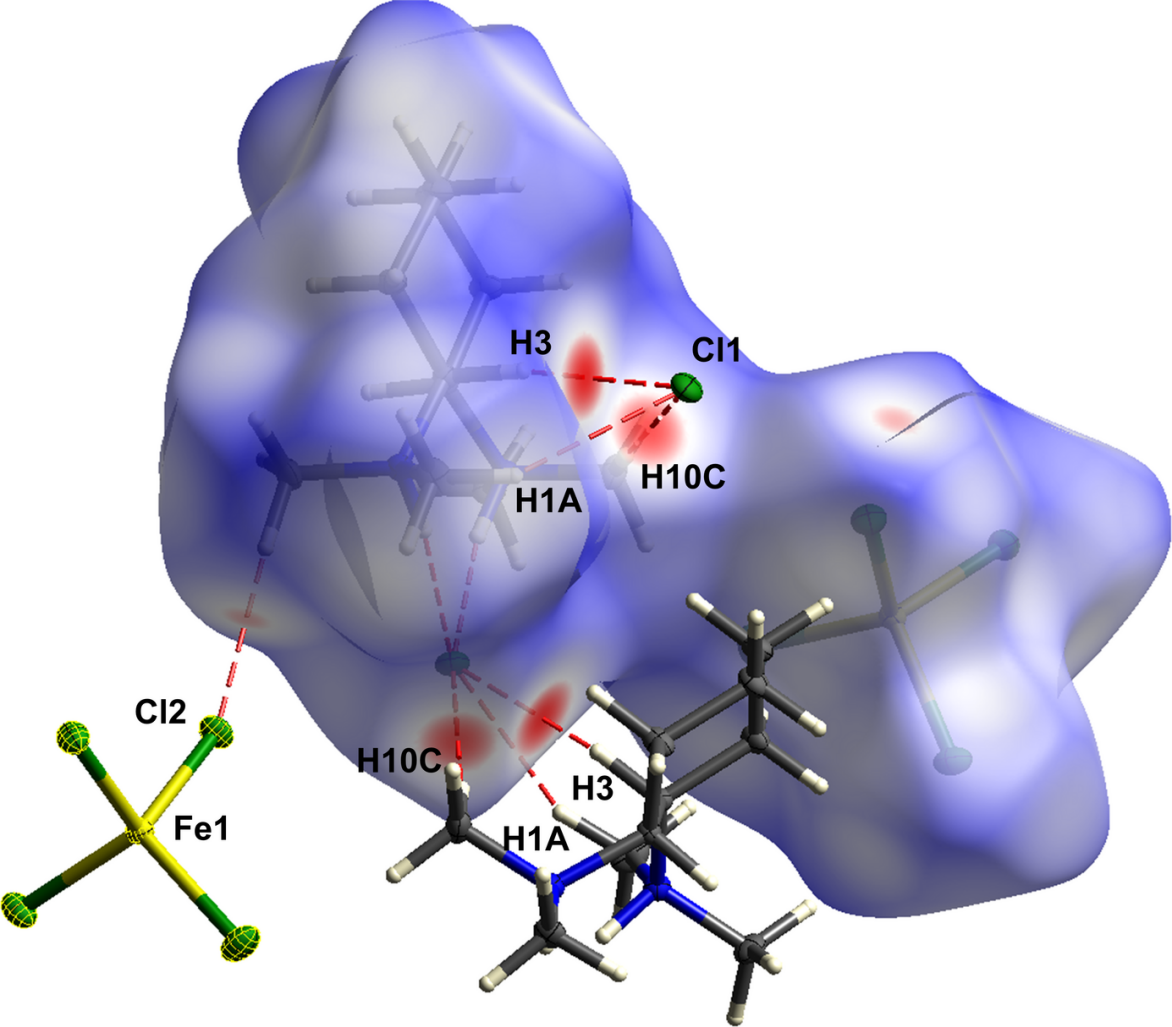
Three-dimensional Hirshfeld surface of **1a** mapped over *d*_norm_ with external fragments and atom labeling (rescale surface property: −0.2124 − 1.4372).

To better understand the supra­molecular inter­actions, a Hirshfeld surface analysis was performed for compound **1a**. In Fig. 3 ▸, the Hirshfeld surface generated by *CrystalExplorer21* (Spackman *et al.*, 2021 ▸) is mapped over *d_norm_* (Spackman & Jayatilaka, 2009 ▸) and red dots are used to represent close contacts between the hydrogen atoms H3, H1*A* and H10*C* with the chloride anion Cl1′. The following figure (Fig. 4 ▸) shows the Hirshfeld surface with external fragments.

**Figure 5:**
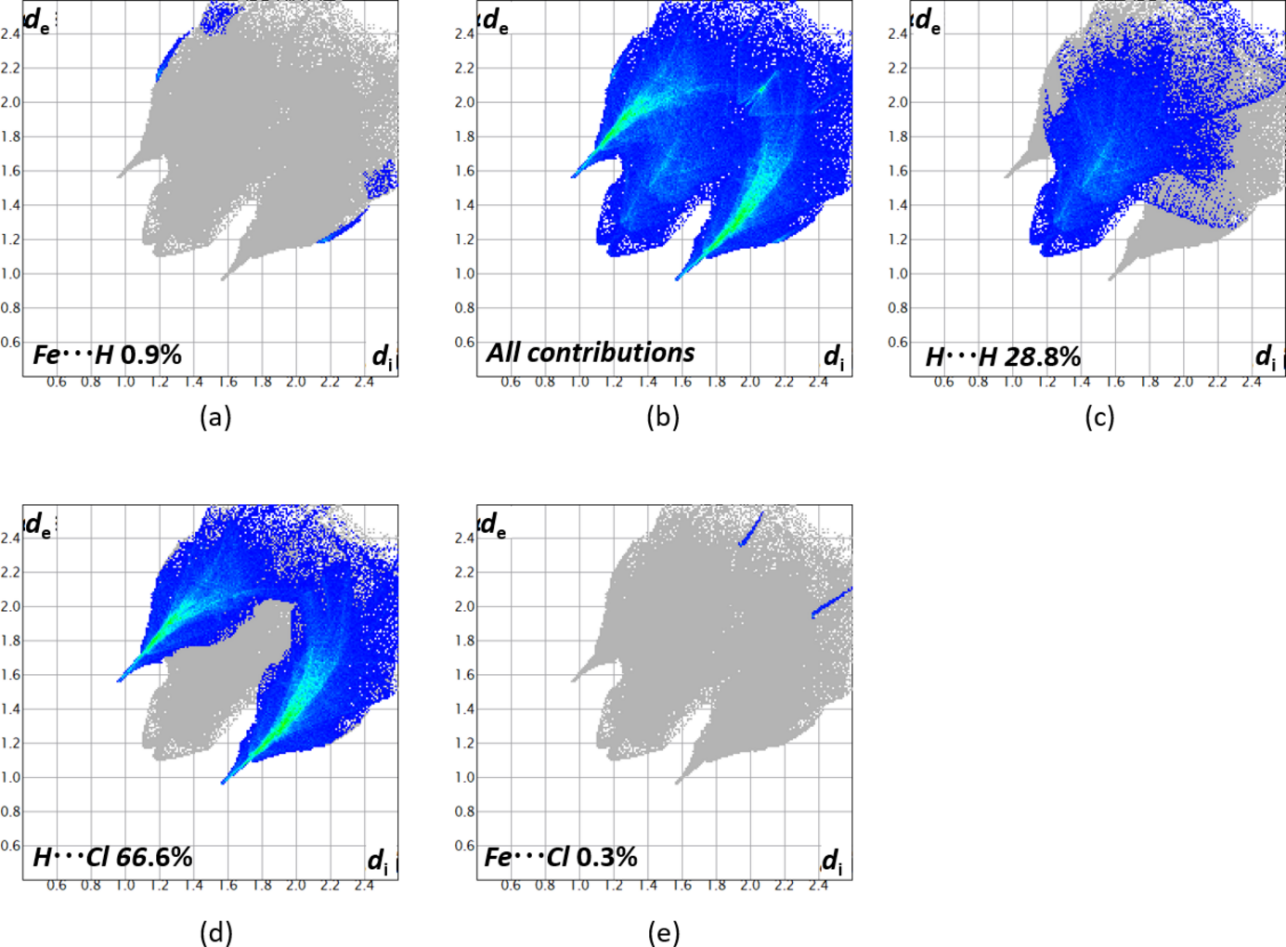
Two-dimensional fingerprint plots for **1a** showing (*b*) all inter­actions, and (*a*) and (*c*)–(*d*) delineated into contributions from other contacts (blue areas) [*d*_e_ and *d*_i_ represent the distances from a point on the Hirshfeld surface to the nearest atoms outside (external) and inside (inter­nal) the surface, respectively].

For further exploration of the inter­molecular inter­actions, two-dimensional fingerprint plots (McKinnon *et al.*, 2007 ▸) were generated as shown in Fig. 5 ▸. The H⋯Cl inter­action with a contribution of 66.6% has the biggest impact on the packing in the solid state as well as the H⋯H bonds with 28.8%. Fe⋯H inter­actions with 0.9% and Cl⋯Fe inter­actions with 0.3% are less impactful in comparison.

**Figure 6:**
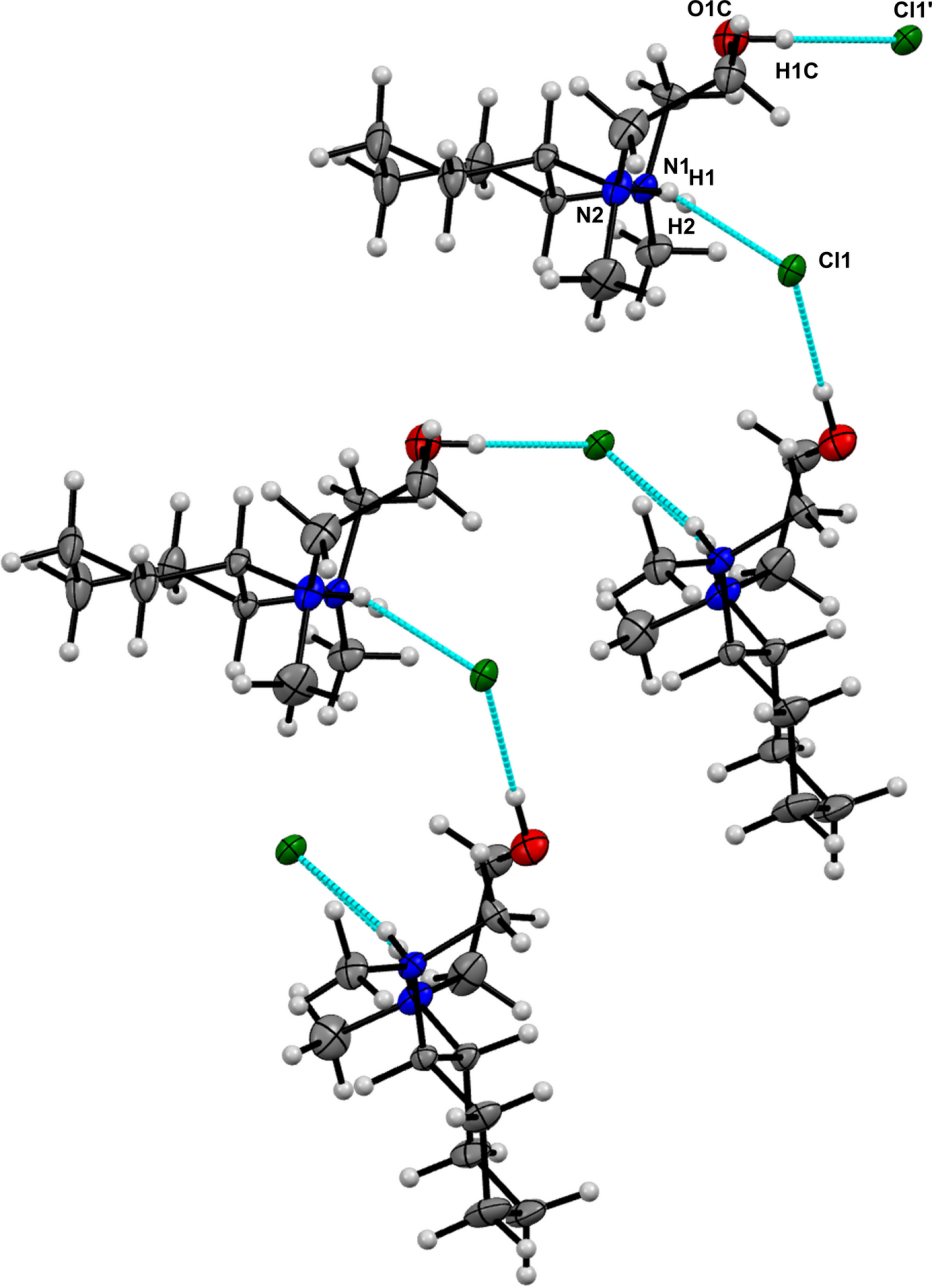
The supra­molecular structure of compound **2a** with the formed hydrogen bonds and the atom labelling of parts of inter­est. The disorder with the lower occupancy (O1*A*, H1*A*) was omitted for clarity. Hydrogen bonds are depicted by dashed lines.

In contrast, compound **2a** forms O—H⋯Cl hydrogen bonds. These inter­actions connect two different moieties of compound **2a** by a chloride, resulting in a supra­molecular zigzag structure in the solid state, as shown in Fig. 6 ▸. They show distances of 2.43 Å (O1*C*—H1*C*⋯Cl1′) and the N2—H2⋯Cl1 distance is measured at 2.357 (3) Å, while the N1—H1⋯Cl1 distance is 2.221 (3) Å. In Fig. 6 ▸ the disorder with the lower occupancy is omitted for clarity, but shows the O1*A*—H1*A*⋯Cl1 hydrogen bond with a distance of 2.22 Å.

## 4. Database survey

A search of the Cambridge Structural Database (CSD version 5.43, November 2021; Groom *et al.*, 2016 ▸) for structures containing (*R*,*R*)-TMCDA and its ethanol derivative both doubly protonated leads to three relevant and similar structures in common CUZZEC, CUZZIG (Duesler *et al.*, 1985 ▸) and POMMIO (Lian *et al.*, 2009 ▸). In these three cases, the amine substituents are –CH_2_COOH fragments. Also, in all three cases there is a single anion, being either CdCl_4_^2−^, PdCl_4_^2−^ or PtCl_4_^2−^ and none of the compounds presents a space group that matches **1a** or **2a**. Another search of structures involving non-protonated (*R*,*R*)-TMCDA leads to several related structures, some of them being: FEJFAD, FEJFIL and FEJFOR (Eckert *et al.*, 2013 ▸), KOBCAH (Eckert *et al.*, 2014 ▸) and LECRUI (Eckert *et al.*, 2012 ▸). FEJFAD and LECRUI show the same space group as **2a** and KOBCAH with **1a**. However, all the latter structures show direct coordination to the metal unlike compounds **1a** and **2a**.

## 5. Synthesis and crystallization

**Figure 7:**
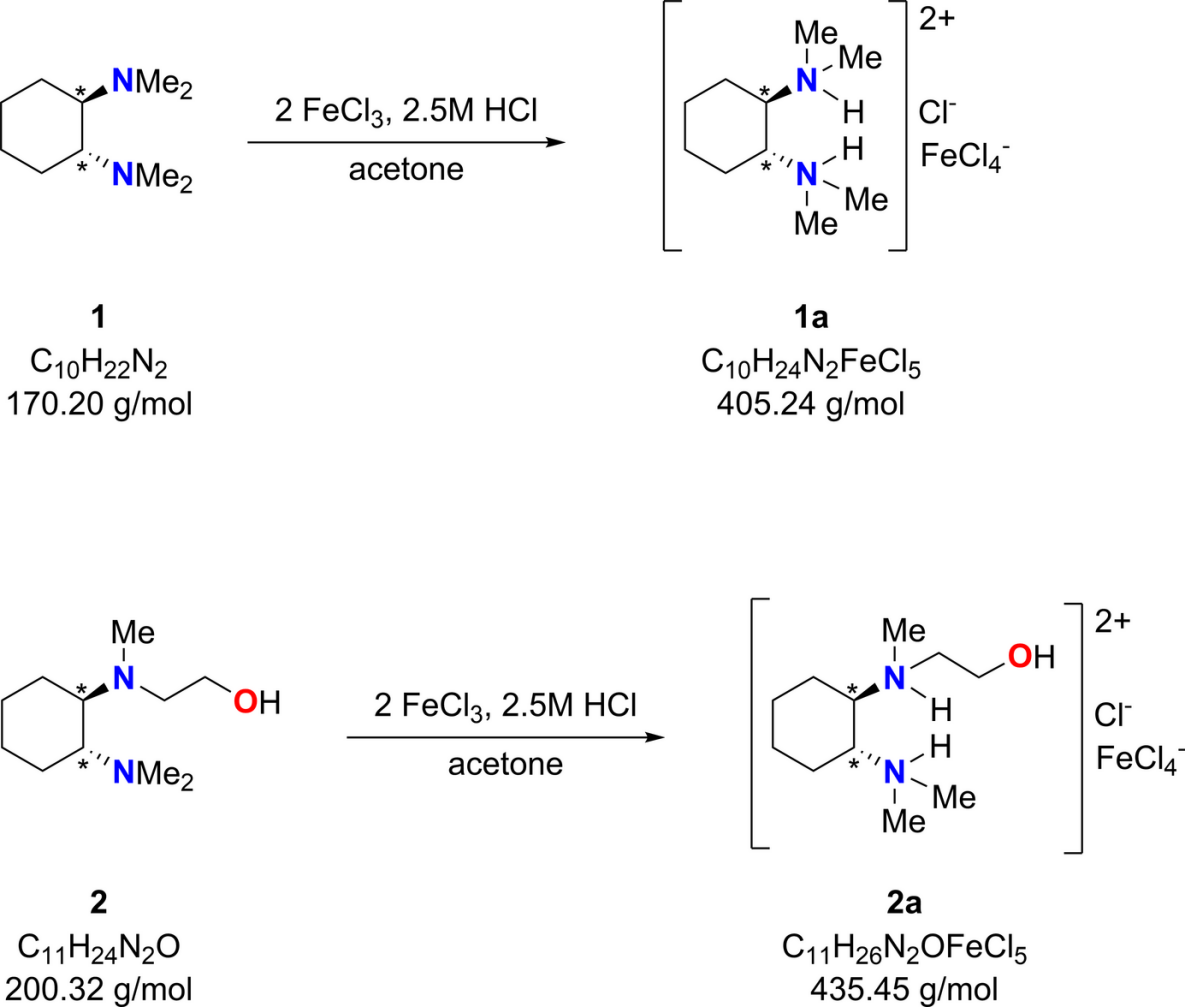
The synthesis of the title compounds.

The syntheses of compounds **1a** and **2a** were conducted according to the previously established procedure (Gessner, 2009 ▸), which involves the mixing of equimolar amounts of FeCl_3_·H_2_O with (*R*,*R*)-TMCDA (**1**) and with 2-{[(1*R*,2*R*)-2-(di­methyl­amino)­cyclo­hex­yl](meth­yl)amino}­ethan-1-ol (**2**), respectively, to yield each compound. In both instances, the reactions were performed in a 3:2 mixture of acetone and 2.5 *M* of HCl at room temperature (see Fig. 7 ▸). Upon complete dissolution of the reactants, a homogeneous yellow solution was obtained, which was then allowed to stand at room temperature until complete evaporation of the solvent. After one week, yellow crystals were formed; specifically, needle-shaped crystals were obtained for compound **1a**, while prism-shaped crystals were obtained for compound **2a**.

## 6. Refinement

Crystal data, data collection and structure refinement details are summarized in Table 3 ▸. Hydrogen atoms except for the protons attached to the nitro­gen and oxygen atoms in both structures were positioned geometrically (C—H = 0.95–1.00 Å) and were refined using a riding model, with *U*_iso_(H) = 1.2*U*_eq_(C) for CH_2_ hydrogen atoms and *U*_iso_(H) = 1.5*U*_eq_(C) for CH_3_ hydrogen atoms.

## Supplementary Material

Crystal structure: contains datablock(s) 2a, 1a. DOI: 10.1107/S2056989025003019/zn2042sup1.cif

Structure factors: contains datablock(s) 2a. DOI: 10.1107/S2056989025003019/zn20422asup3.hkl

Structure factors: contains datablock(s) 1a. DOI: 10.1107/S2056989025003019/zn20421asup2.hkl

CCDC references: 2440777, 2440778

Additional supporting information:  crystallographic information; 3D view; checkCIF report

## Figures and Tables

**Table 1:** Hydrogen-bond geometry (Å, °) for **1a**[Chem scheme1]

*D*—H⋯*A*	*D*—H	H⋯*A*	*D*⋯*A*	*D*—H⋯*A*
N1—H1⋯Cl1	0.891 (19)	2.133 (19)	3.0189 (11)	172.7 (19)
N2—H2⋯Cl1	0.900 (18)	2.135 (17)	3.0208 (10)	165 (14)

**Table 2:** Hydrogen-bond geometry (Å, °) for **2a**[Chem scheme1]

*D*—H⋯*A*	*D*—H	H⋯*A*	*D*⋯*A*	*D*—H⋯*A*
N1—H1⋯Cl1	0.855 (3)	2.221 (3)	3.0603 (14)	167 (3)
N2—H2⋯Cl1	0.878 (3)	2.357 (3)	3.1698 (16)	154 (3)
O1*C*—H1*C*⋯Cl1^i^	0.84	2.43	3.229 (3)	159

Symmetry code: (i) 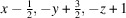.

**Table 3:** Experimental details

	**1a**	**2a**
Crystal data		
Chemical formula	(C_10_H_24_N_2_)[FeCl_4_]Cl	(C_11_H_26_N_2_O)[FeCl_4_]Cl
*M*_r_	405.41	435.44
Crystal system, space group	Monoclinic, *P*2_1_	Orthorhombic, *P*2_1_2_1_2_1_
Temperature (K)	100	100
*a*, *b*, *c* (Å)	10.2322 (3), 9.2378 (5), 10.7384 (4)	7.6070 (5), 11.5870 (6), 21.8705 (13)
α, β, γ (°)	90, 116.797 (1), 90	90, 90, 90
*V* (Å^3^)	906.02 (7)	1927.7 (2)
*Z*	2	4
Radiation type	Mo *K*α	Mo *K*α
μ (mm^−1^)	1.56	1.47
Crystal size (mm)	0.50 × 0.43 × 0.38	0.8 × 0.43 × 0.18

Data collection		
Diffractometer	Bruker D8 VENTURE area detector	Bruker APEXII CCD
Absorption correction	–	Multi-scan (*SADABS*; Krause *et al.*, 2015 ▸)
*T*_min_, *T*_max_	–	0.472, 0.567
No. of measured, independent and observed [*I* > 2σ(*I*)] reflections	69005, 8763, 8442	144077, 8488, 8082
*R*_int_	0.030	0.046
(sin θ/λ)_max_ (Å^−1^)	0.834	0.807

Refinement		
*R*[*F*^2^ > 2σ(*F*^2^)], *wR*(*F*^2^), *S*	0.018, 0.037, 1.06	0.026, 0.063, 1.07
No. of reflections	8763	8488
No. of parameters	175	231
No. of restraints	1	1
H-atom treatment	H atoms treated by a mixture of independent and constrained refinement	H atoms treated by a mixture of independent and constrained refinement
Δρ_max_, Δρ_min_ (e Å^−3^)	0.23, −0.34	0.61, −0.48
Absolute structure	Flack *x* determined using 3826 quotients [(*I*^+^)−(*I*^−^)]/[(*I*^+^)+(*I*^−^)] (Parsons *et al.*, 2013 ▸)	Flack *x* determined using 3373 quotients [(*I*^+^)−(*I*^−^)]/[(*I*^+^)+(*I*^−^)] (Parsons *et al.*, 2013 ▸)
Absolute structure parameter	−0.009 (3)	−0.013 (4)

Computer programs: *APEX2* and *SAINT* (Bruker, 2016 ▸), *SHELXT* (Sheldrick, 2015*a* ▸), *SHELXL* (Sheldrick, 2015*b* ▸) and *OLEX2* (Dolomanov *et al.*, 2009 ▸).

## supplementary crystallographic information

### (R,R)-N1-(2-Hydroxyethyl)-N1,N2,N2-trimethylcyclohexane-1,2-bis(aminium) tetrachloridoferrate chloride (2a) Crystal data

**Table:**

(C_11_H_26_N_2_O)[FeCl_4_]Cl	*D*_x_ = 1.500 Mg m^−^^3^
*M**_r_* = 435.44	Mo *K*α radiation, λ = 0.71073 Å
Orthorhombic, *P*2_1_2_1_2_1_	Cell parameters from 9581 reflections
*a* = 7.6070 (5) Å	θ = 2.6–34.8°
*b* = 11.5870 (6) Å	µ = 1.47 mm^−^^1^
*c* = 21.8705 (13) Å	*T* = 100 K
*V* = 1927.7 (2) Å^3^	Needle, yellow
*Z* = 4	0.8 × 0.43 × 0.18 mm
*F*(000) = 900	

### (R,R)-N1-(2-Hydroxyethyl)-N1,N2,N2-trimethylcyclohexane-1,2-bis(aminium) tetrachloridoferrate chloride (2a) Data collection

**Table:**

Bruker APEXII CCD diffractometer	8488 independent reflections
Radiation source: microfocus sealed X-ray tube, Incoatec Iµs	8082 reflections with *I* > 2σ(*I*)
HELIOS mirror optics monochromator	*R*_int_ = 0.046
Detector resolution: 10.4167 pixels mm^-1^	θ_max_ = 35.0°, θ_min_ = 1.9°
φ and ω scans	*h* = −12→12
Absorption correction: multi-scan (SADABS; Krause *et al.*, 2015)	*k* = −18→18
*T*_min_ = 0.472, *T*_max_ = 0.567	*l* = −35→35
144077 measured reflections	

### (R,R)-N1-(2-Hydroxyethyl)-N1,N2,N2-trimethylcyclohexane-1,2-bis(aminium) tetrachloridoferrate chloride (2a) Refinement

**Table:**

Refinement on *F*^2^	Hydrogen site location: mixed
Least-squares matrix: full	H atoms treated by a mixture of independent and constrained refinement
*R*[*F*^2^ > 2σ(*F*^2^)] = 0.026	*w* = 1/[σ^2^(*F*_o_^2^) + (0.0263*P*)^2^ + 0.5573*P*] where *P* = (*F*_o_^2^ + 2*F*_c_^2^)/3
*wR*(*F*^2^) = 0.063	(Δ/σ)_max_ = 0.001
*S* = 1.07	Δρ_max_ = 0.61 e Å^−^^3^
8488 reflections	Δρ_min_ = −0.48 e Å^−^^3^
231 parameters	Absolute structure: Flack *x* determined using 3373 quotients [(*I*^+^)-(*I*^-^)]/[(*I*^+^)+(*I*^-^)] (Parsons *et al.*, 2013)
1 restraint	Absolute structure parameter: −0.013 (4)
Primary atom site location: dual	

### (R,R)-N1-(2-Hydroxyethyl)-N1,N2,N2-trimethylcyclohexane-1,2-bis(aminium) tetrachloridoferrate chloride (2a) Special details

**Table:**

Geometry. All esds (except the esd in the dihedral angle between two l.s. planes) are estimated using the full covariance matrix. The cell esds are taken into account individually in the estimation of esds in distances, angles and torsion angles; correlations between esds in cell parameters are only used when they are defined by crystal symmetry. An approximate (isotropic) treatment of cell esds is used for estimating esds involving l.s. planes.

### (R,R)-N1-(2-Hydroxyethyl)-N1,N2,N2-trimethylcyclohexane-1,2-bis(aminium) tetrachloridoferrate chloride (2a) Fractional atomic coordinates and isotropic or equivalent isotropic displacement parameters (Å^2^)

**Table:**

	*x*	*y*	*z*	*U*_iso_*/*U*_eq_	Occ. (<1)
Fe1	0.38908 (3)	0.32831 (2)	0.64131 (2)	0.02140 (5)	
Cl1	0.32469 (6)	0.71475 (4)	0.52176 (2)	0.02393 (7)	
Cl5	0.40990 (7)	0.16915 (4)	0.69535 (2)	0.03204 (10)	
Cl4	0.63253 (7)	0.34762 (4)	0.58818 (2)	0.02908 (9)	
Cl3	0.36082 (7)	0.47621 (5)	0.70394 (3)	0.03907 (12)	
Cl2	0.16007 (8)	0.31529 (4)	0.58067 (3)	0.04136 (13)	
N1	0.36655 (19)	0.80677 (11)	0.39141 (6)	0.0176 (2)	
C4	0.49489 (19)	0.73202 (12)	0.35567 (7)	0.0167 (2)	
H4	0.610719	0.734966	0.377232	0.020*	
C9	0.4413 (2)	0.60442 (13)	0.35010 (7)	0.0190 (3)	
H9	0.330578	0.600659	0.325512	0.023*	
N2	0.4066 (2)	0.54577 (12)	0.41140 (7)	0.0229 (3)	
C2A	0.1906 (10)	0.8308 (10)	0.3630 (5)	0.0204 (19)	0.6
H2AA	0.204658	0.886344	0.329535	0.031*	0.6
H2AB	0.111296	0.863005	0.393931	0.031*	0.6
H2AC	0.140939	0.758896	0.346966	0.031*	0.6
C8	0.5840 (3)	0.53894 (15)	0.31517 (9)	0.0285 (4)	
H8A	0.695235	0.542237	0.338627	0.034*	
H8B	0.549412	0.456878	0.311433	0.034*	
C5	0.5215 (3)	0.78172 (17)	0.29127 (8)	0.0283 (4)	
H5A	0.409277	0.777667	0.268459	0.034*	
H5B	0.555774	0.863928	0.294475	0.034*	
C7	0.6146 (3)	0.58882 (18)	0.25142 (9)	0.0329 (4)	
H7A	0.710524	0.546051	0.230899	0.040*	
H7B	0.506586	0.580115	0.226574	0.040*	
C10	0.5676 (3)	0.51474 (19)	0.44679 (11)	0.0380 (5)	
H10A	0.632514	0.454339	0.425011	0.057*	
H10B	0.533646	0.486423	0.487323	0.057*	
H10C	0.642387	0.583097	0.451261	0.057*	
C6	0.6630 (3)	0.71594 (19)	0.25612 (10)	0.0360 (5)	
H6A	0.675303	0.748989	0.214598	0.043*	
H6B	0.777242	0.723985	0.277372	0.043*	
C3	0.4494 (3)	0.92074 (14)	0.40779 (9)	0.0261 (3)	
H3A	0.566736	0.907371	0.424913	0.039*	
H3B	0.376186	0.960350	0.438098	0.039*	
H3C	0.459308	0.968647	0.371023	0.039*	
C2C	0.3030 (10)	0.4364 (7)	0.4077 (4)	0.0352 (15)	0.6
H2CA	0.353142	0.375708	0.434330	0.042*	0.6
H2CB	0.296099	0.407377	0.365179	0.042*	0.6
O1C	0.0357 (4)	0.5852 (2)	0.40633 (12)	0.0303 (5)	0.6
H1C	−0.026743	0.622278	0.431171	0.045*	0.6
O1A	0.0770 (5)	0.6464 (3)	0.40150 (16)	0.0268 (6)	0.4
H1A	0.015547	0.671492	0.430532	0.040*	0.4
C1C	0.0962 (5)	0.4832 (3)	0.43433 (15)	0.0273 (6)	0.6
H1CA	0.009140	0.421137	0.427170	0.033*	0.6
H1CB	0.103627	0.496020	0.479006	0.033*	0.6
C1A	0.0736 (6)	0.7274 (4)	0.3522 (2)	0.0242 (7)	0.4
H1AA	−0.047730	0.756592	0.346835	0.029*	0.4
H1AB	0.108667	0.688030	0.313934	0.029*	0.4
H2	0.351 (4)	0.594 (2)	0.4354 (13)	0.033 (7)*	
C2D	0.2584 (14)	0.4516 (10)	0.3996 (6)	0.0256 (17)	0.4
H2DA	0.230905	0.448666	0.355885	0.038*	0.4
H2DB	0.152364	0.472246	0.422608	0.038*	0.4
H2DC	0.300608	0.375886	0.413154	0.038*	0.4
H1	0.342 (4)	0.774 (2)	0.4253 (13)	0.037 (7)*	
C2B	0.1897 (17)	0.8228 (16)	0.3631 (7)	0.028 (4)	0.4
H2BA	0.125018	0.878280	0.389230	0.034*	0.4
H2BB	0.208584	0.861386	0.323279	0.034*	0.4

### (R,R)-N1-(2-Hydroxyethyl)-N1,N2,N2-trimethylcyclohexane-1,2-bis(aminium) tetrachloridoferrate chloride (2a) Atomic displacement parameters (Å^2^)

**Table:**

	*U*^11^	*U*^22^	*U*^33^	*U*^12^	*U*^13^	*U*^23^
Fe1	0.02100 (10)	0.02097 (10)	0.02223 (10)	−0.00210 (8)	−0.00138 (8)	−0.00180 (8)
Cl1	0.02587 (17)	0.03036 (18)	0.01556 (13)	−0.00029 (15)	0.00178 (13)	−0.00037 (13)
Cl5	0.0315 (2)	0.0350 (2)	0.02956 (19)	−0.00769 (18)	0.00039 (16)	0.01233 (18)
Cl4	0.0340 (2)	0.02166 (16)	0.03163 (19)	0.00026 (15)	0.01043 (17)	0.00573 (14)
Cl3	0.0339 (2)	0.0387 (2)	0.0446 (3)	−0.0104 (2)	0.0089 (2)	−0.0217 (2)
Cl2	0.0391 (3)	0.0284 (2)	0.0566 (3)	0.00584 (19)	−0.0261 (2)	−0.0091 (2)
N1	0.0228 (6)	0.0148 (5)	0.0151 (5)	0.0018 (4)	0.0004 (4)	−0.0001 (4)
C4	0.0181 (6)	0.0152 (5)	0.0168 (6)	−0.0002 (4)	0.0009 (5)	−0.0016 (5)
C9	0.0223 (6)	0.0177 (6)	0.0171 (6)	−0.0033 (5)	0.0017 (5)	−0.0042 (5)
N2	0.0330 (7)	0.0138 (5)	0.0220 (6)	−0.0028 (5)	0.0049 (6)	−0.0017 (5)
C2A	0.018 (3)	0.0139 (19)	0.030 (4)	0.0001 (17)	−0.006 (2)	0.0035 (19)
C8	0.0345 (9)	0.0208 (7)	0.0303 (8)	0.0009 (6)	0.0101 (7)	−0.0093 (6)
C5	0.0386 (10)	0.0254 (8)	0.0209 (7)	0.0002 (7)	0.0093 (7)	0.0029 (6)
C7	0.0374 (10)	0.0344 (9)	0.0270 (8)	−0.0042 (8)	0.0141 (8)	−0.0121 (7)
C10	0.0487 (13)	0.0285 (9)	0.0368 (10)	0.0111 (9)	−0.0049 (9)	0.0087 (8)
C6	0.0425 (11)	0.0328 (9)	0.0326 (9)	−0.0076 (9)	0.0215 (9)	−0.0044 (7)
C3	0.0321 (9)	0.0164 (6)	0.0299 (8)	0.0006 (6)	−0.0035 (7)	−0.0057 (6)
C2C	0.053 (5)	0.024 (3)	0.029 (2)	−0.019 (3)	0.004 (3)	−0.0007 (19)
O1C	0.0374 (13)	0.0201 (10)	0.0335 (12)	0.0007 (9)	0.0022 (10)	−0.0058 (9)
O1A	0.0258 (15)	0.0288 (17)	0.0258 (15)	0.0005 (13)	0.0024 (11)	−0.0015 (13)
C1C	0.0348 (15)	0.0202 (11)	0.0269 (13)	−0.0052 (11)	0.0076 (12)	−0.0023 (10)
C1A	0.0215 (17)	0.0282 (19)	0.0229 (17)	0.0013 (14)	−0.0015 (14)	−0.0029 (15)
C2D	0.030 (4)	0.011 (2)	0.035 (4)	−0.003 (2)	0.006 (3)	−0.001 (2)
C2B	0.027 (6)	0.035 (7)	0.023 (5)	0.008 (4)	0.011 (4)	−0.002 (4)

### (R,R)-N1-(2-Hydroxyethyl)-N1,N2,N2-trimethylcyclohexane-1,2-bis(aminium) tetrachloridoferrate chloride (2a) Geometric parameters (Å, º)

**Table:**

Fe1—Cl5	2.1962 (5)	C7—H7A	0.9900
Fe1—Cl4	2.1976 (5)	C7—H7B	0.9900
Fe1—Cl3	2.2044 (5)	C7—C6	1.522 (3)
Fe1—Cl2	2.1946 (6)	C10—H10A	0.9800
N1—C4	1.521 (2)	C10—H10B	0.9800
N1—C2A	1.502 (7)	C10—H10C	0.9800
N1—C3	1.507 (2)	C6—H6A	0.9900
N1—H1	0.86 (3)	C6—H6B	0.9900
N1—C2B	1.492 (13)	C3—H3A	0.9800
C4—H4	1.0000	C3—H3B	0.9800
C4—C9	1.539 (2)	C3—H3C	0.9800
C4—C5	1.535 (2)	C2C—H2CA	0.9900
C9—H9	1.0000	C2C—H2CB	0.9900
C9—N2	1.526 (2)	C2C—C1C	1.763 (9)
C9—C8	1.529 (2)	O1C—H1C	0.8400
N2—C10	1.493 (3)	O1C—C1C	1.408 (4)
N2—C2C	1.494 (9)	O1A—H1A	0.8400
N2—H2	0.88 (3)	O1A—C1A	1.429 (6)
N2—C2D	1.590 (13)	C1C—H1CA	0.9900
C2A—H2AA	0.9800	C1C—H1CB	0.9900
C2A—H2AB	0.9800	C1A—H1AA	0.9900
C2A—H2AC	0.9800	C1A—H1AB	0.9900
C8—H8A	0.9900	C1A—C2B	1.435 (18)
C8—H8B	0.9900	C2D—H2DA	0.9800
C8—C7	1.527 (3)	C2D—H2DB	0.9800
C5—H5A	0.9900	C2D—H2DC	0.9800
C5—H5B	0.9900	C2B—H2BA	0.9900
C5—C6	1.526 (3)	C2B—H2BB	0.9900
			
Cl5—Fe1—Cl4	108.02 (2)	C8—C7—H7B	109.7
Cl5—Fe1—Cl3	108.99 (3)	H7A—C7—H7B	108.2
Cl4—Fe1—Cl3	109.37 (2)	C6—C7—C8	109.97 (16)
Cl2—Fe1—Cl5	108.95 (2)	C6—C7—H7A	109.7
Cl2—Fe1—Cl4	110.88 (3)	C6—C7—H7B	109.7
Cl2—Fe1—Cl3	110.58 (2)	N2—C10—H10A	109.5
C4—N1—H1	109 (2)	N2—C10—H10B	109.5
C2A—N1—C4	117.7 (4)	N2—C10—H10C	109.5
C2A—N1—C3	108.0 (4)	H10A—C10—H10B	109.5
C2A—N1—H1	104 (2)	H10A—C10—H10C	109.5
C3—N1—C4	110.65 (13)	H10B—C10—H10C	109.5
C3—N1—H1	106.0 (19)	C5—C6—H6A	109.6
C2B—N1—C4	115.9 (6)	C5—C6—H6B	109.6
C2B—N1—C3	111.5 (7)	C7—C6—C5	110.28 (17)
C2B—N1—H1	103 (2)	C7—C6—H6A	109.6
N1—C4—H4	107.7	C7—C6—H6B	109.6
N1—C4—C9	114.68 (12)	H6A—C6—H6B	108.1
N1—C4—C5	110.03 (13)	N1—C3—H3A	109.5
C9—C4—H4	107.7	N1—C3—H3B	109.5
C5—C4—H4	107.7	N1—C3—H3C	109.5
C5—C4—C9	108.84 (13)	H3A—C3—H3B	109.5
C4—C9—H9	107.9	H3A—C3—H3C	109.5
N2—C9—C4	113.85 (12)	H3B—C3—H3C	109.5
N2—C9—H9	107.9	N2—C2C—H2CA	111.6
N2—C9—C8	109.91 (14)	N2—C2C—H2CB	111.6
C8—C9—C4	109.14 (13)	N2—C2C—C1C	101.1 (5)
C8—C9—H9	107.9	H2CA—C2C—H2CB	109.4
C9—N2—H2	108.7 (18)	C1C—C2C—H2CA	111.6
C9—N2—C2D	106.6 (5)	C1C—C2C—H2CB	111.6
C10—N2—C9	114.90 (15)	C1C—O1C—H1C	109.5
C10—N2—C2C	104.9 (3)	C1A—O1A—H1A	109.5
C10—N2—H2	103.8 (18)	C2C—C1C—H1CA	108.8
C10—N2—C2D	120.0 (3)	C2C—C1C—H1CB	108.8
C2C—N2—C9	114.9 (4)	O1C—C1C—C2C	113.9 (3)
C2C—N2—H2	109.0 (18)	O1C—C1C—H1CA	108.8
C2D—N2—H2	101.4 (19)	O1C—C1C—H1CB	108.8
N1—C2A—H2AA	109.5	H1CA—C1C—H1CB	107.7
N1—C2A—H2AB	109.5	O1A—C1A—H1AA	109.3
N1—C2A—H2AC	109.5	O1A—C1A—H1AB	109.3
H2AA—C2A—H2AB	109.5	O1A—C1A—C2B	111.7 (7)
H2AA—C2A—H2AC	109.5	H1AA—C1A—H1AB	107.9
H2AB—C2A—H2AC	109.5	C2B—C1A—H1AA	109.3
C9—C8—H8A	109.2	C2B—C1A—H1AB	109.3
C9—C8—H8B	109.2	N2—C2D—H2DA	109.5
H8A—C8—H8B	107.9	N2—C2D—H2DB	109.5
C7—C8—C9	112.10 (16)	N2—C2D—H2DC	109.5
C7—C8—H8A	109.2	H2DA—C2D—H2DB	109.5
C7—C8—H8B	109.2	H2DA—C2D—H2DC	109.5
C4—C5—H5A	109.3	H2DB—C2D—H2DC	109.5
C4—C5—H5B	109.3	N1—C2B—H2BA	106.9
H5A—C5—H5B	108.0	N1—C2B—H2BB	106.9
C6—C5—C4	111.58 (16)	C1A—C2B—N1	121.8 (12)
C6—C5—H5A	109.3	C1A—C2B—H2BA	106.9
C6—C5—H5B	109.3	C1A—C2B—H2BB	106.9
C8—C7—H7A	109.7	H2BA—C2B—H2BB	106.7
			
N1—C4—C9—N2	−54.93 (18)	C2A—N1—C4—C5	53.8 (5)
N1—C4—C9—C8	−178.13 (14)	C8—C9—N2—C10	46.55 (19)
N1—C4—C5—C6	174.42 (16)	C8—C9—N2—C2C	−75.3 (4)
C4—N1—C2B—C1A	61.9 (12)	C8—C9—N2—C2D	−89.0 (4)
C4—C9—N2—C10	−76.24 (18)	C8—C7—C6—C5	−55.7 (3)
C4—C9—N2—C2C	161.9 (3)	C5—C4—C9—N2	−178.65 (14)
C4—C9—N2—C2D	148.2 (4)	C5—C4—C9—C8	58.15 (18)
C4—C9—C8—C7	−58.7 (2)	C10—N2—C2C—C1C	128.1 (3)
C4—C5—C6—C7	58.1 (2)	C3—N1—C4—C9	165.95 (13)
C9—C4—C5—C6	−59.1 (2)	C3—N1—C4—C5	−70.97 (17)
C9—N2—C2C—C1C	−104.8 (4)	C3—N1—C2B—C1A	−170.3 (8)
C9—C8—C7—C6	57.3 (2)	O1A—C1A—C2B—N1	40.0 (12)
N2—C9—C8—C7	175.82 (15)	C2B—N1—C4—C9	−65.9 (8)
N2—C2C—C1C—O1C	48.6 (5)	C2B—N1—C4—C5	57.2 (8)
C2A—N1—C4—C9	−69.3 (5)		

### (R,R)-N1-(2-Hydroxyethyl)-N1,N2,N2-trimethylcyclohexane-1,2-bis(aminium) tetrachloridoferrate chloride (2a) Hydrogen-bond geometry (Å, º)

**Table:**

*D*—H···*A*	*D*—H	H···*A*	*D*···*A*	*D*—H···*A*
N1—H1···Cl1	0.855 (3)	2.221 (3)	3.0603 (14)	167 (3)
N2—H2···Cl1	0.878 (3)	2.357 (3)	3.1698 (16)	154 (3)
O1*C*—H1*C*···Cl1^i^	0.84	2.43	3.229 (3)	159

Symmetry code: (i) *x*−1/2, −*y*+3/2, −*z*+1.

### (R,R)-N1,N1,N2,N2-Tetramethylcyclohexane-1,2-bis(aminium) tetrachloridoferrate chloride (1a) Crystal data

**Table:**

(C_10_H_24_N_2_)[FeCl_4_]Cl	*F*(000) = 418
*M**_r_* = 405.41	*D*_x_ = 1.486 Mg m^−^^3^
Monoclinic, *P*2_1_	Mo *K*α radiation, λ = 0.71073 Å
*a* = 10.2322 (3) Å	Cell parameters from 1350 reflections
*b* = 9.2378 (5) Å	θ = 4.3–31.7°
*c* = 10.7384 (4) Å	µ = 1.56 mm^−^^1^
β = 116.797 (1)°	*T* = 100 K
*V* = 906.02 (7) Å^3^	Prism, yellow
*Z* = 2	0.50 × 0.43 × 0.38 mm

### (R,R)-N1,N1,N2,N2-Tetramethylcyclohexane-1,2-bis(aminium) tetrachloridoferrate chloride (1a) Data collection

**Table:**

Bruker D8 VENTURE area detector diffractometer	8442 reflections with *I* > 2σ(*I*)
Radiation source: microfocus sealed X-ray tube, Incoatec Iµs	*R*_int_ = 0.030
HELIOS mirror optics monochromator	θ_max_ = 36.3°, θ_min_ = 2.1°
Detector resolution: 10.4167 pixels mm^-1^	*h* = −13→17
ω and φ scans	*k* = −15→15
69005 measured reflections	*l* = −17→17
8763 independent reflections	

### (R,R)-N1,N1,N2,N2-Tetramethylcyclohexane-1,2-bis(aminium) tetrachloridoferrate chloride (1a) Refinement

**Table:**

Refinement on *F*^2^	Hydrogen site location: mixed
Least-squares matrix: full	H atoms treated by a mixture of independent and constrained refinement
*R*[*F*^2^ > 2σ(*F*^2^)] = 0.018	*w* = 1/[σ^2^(*F*_o_^2^) + (0.0156*P*)^2^ + 0.078*P*] where *P* = (*F*_o_^2^ + 2*F*_c_^2^)/3
*wR*(*F*^2^) = 0.037	(Δ/σ)_max_ = 0.001
*S* = 1.06	Δρ_max_ = 0.23 e Å^−^^3^
8763 reflections	Δρ_min_ = −0.33 e Å^−^^3^
175 parameters	Absolute structure: Flack *x* determined using 3826 quotients [(*I*^+^)-(*I*^-^)]/[(*I*^+^)+(*I*^-^)] (Parsons *et al.*, 2013)
1 restraint	Absolute structure parameter: −0.009 (3)
Primary atom site location: dual	

### (R,R)-N1,N1,N2,N2-Tetramethylcyclohexane-1,2-bis(aminium) tetrachloridoferrate chloride (1a) Special details

**Table:**

Geometry. All esds (except the esd in the dihedral angle between two l.s. planes) are estimated using the full covariance matrix. The cell esds are taken into account individually in the estimation of esds in distances, angles and torsion angles; correlations between esds in cell parameters are only used when they are defined by crystal symmetry. An approximate (isotropic) treatment of cell esds is used for estimating esds involving l.s. planes.

### (R,R)-N1,N1,N2,N2-Tetramethylcyclohexane-1,2-bis(aminium) tetrachloridoferrate chloride (1a) Fractional atomic coordinates and isotropic or equivalent isotropic displacement parameters (Å^2^)

**Table:**

	*x*	*y*	*z*	*U*_iso_*/*U*_eq_	
Fe1	0.73543 (2)	0.37018 (2)	0.11080 (2)	0.01357 (3)	
Cl2	0.76183 (3)	0.51878 (3)	0.28052 (3)	0.01944 (5)	
Cl5	0.76963 (3)	0.49518 (3)	−0.04548 (3)	0.01960 (5)	
Cl1	0.35677 (3)	0.17053 (3)	0.42055 (3)	0.01882 (5)	
Cl3	0.52216 (3)	0.26207 (4)	0.02175 (3)	0.02597 (6)	
Cl4	0.90709 (3)	0.20084 (3)	0.19738 (3)	0.02248 (5)	
N1	0.28286 (10)	0.44077 (10)	0.53384 (9)	0.01356 (15)	
N2	0.26193 (10)	0.43743 (10)	0.23871 (9)	0.01275 (14)	
C8	0.18173 (11)	0.54564 (11)	0.28693 (10)	0.01174 (15)	
H8	0.0835	0.5042	0.2652	0.014*	
C9	0.17020 (12)	0.38898 (13)	0.09145 (11)	0.01754 (19)	
H9A	0.0722	0.3629	0.0790	0.026*	
H9B	0.2159	0.3046	0.0717	0.026*	
H9C	0.1625	0.4677	0.0274	0.026*	
C7	0.15769 (13)	0.68716 (12)	0.20441 (11)	0.01745 (19)	
H7A	0.2536	0.7251	0.2178	0.021*	
H7B	0.0983	0.6667	0.1038	0.021*	
C3	0.26269 (11)	0.57574 (11)	0.44544 (10)	0.01184 (15)	
H3	0.3623	0.6133	0.4670	0.014*	
C6	0.08042 (13)	0.80253 (12)	0.24935 (12)	0.01837 (19)	
H6A	0.0700	0.8927	0.1958	0.022*	
H6B	−0.0187	0.7685	0.2299	0.022*	
C1	0.40538 (13)	0.45802 (15)	0.67836 (11)	0.0231 (2)	
H1A	0.4946	0.4876	0.6728	0.035*	
H1B	0.4226	0.3657	0.7282	0.035*	
H1C	0.3790	0.5320	0.7284	0.035*	
C5	0.16937 (13)	0.83247 (12)	0.40466 (12)	0.01787 (19)	
H5A	0.1201	0.9077	0.4341	0.021*	
H5B	0.2677	0.8686	0.4238	0.021*	
C10	0.40958 (12)	0.48351 (14)	0.25595 (12)	0.0200 (2)	
H10A	0.3979	0.5602	0.1887	0.030*	
H10B	0.4595	0.4005	0.2396	0.030*	
H10C	0.4679	0.5200	0.3509	0.030*	
C4	0.18391 (12)	0.69326 (12)	0.48655 (11)	0.01606 (18)	
H4A	0.0853	0.6584	0.4681	0.019*	
H4B	0.2394	0.7133	0.5877	0.019*	
C2	0.14826 (12)	0.38899 (13)	0.54129 (12)	0.0194 (2)	
H2A	0.1253	0.4545	0.6005	0.029*	
H2B	0.1651	0.2913	0.5810	0.029*	
H2C	0.0661	0.3872	0.4473	0.029*	
H1	0.3094 (18)	0.366 (2)	0.4978 (18)	0.025 (4)*	
H2	0.2756 (17)	0.3539 (19)	0.2862 (17)	0.022 (4)*	

### (R,R)-N1,N1,N2,N2-Tetramethylcyclohexane-1,2-bis(aminium) tetrachloridoferrate chloride (1a) Atomic displacement parameters (Å^2^)

**Table:**

	*U*^11^	*U*^22^	*U*^33^	*U*^12^	*U*^13^	*U*^23^
Fe1	0.01513 (6)	0.01373 (6)	0.01181 (6)	−0.00141 (5)	0.00605 (5)	−0.00101 (5)
Cl2	0.02272 (11)	0.01915 (11)	0.01510 (10)	0.00063 (9)	0.00734 (9)	−0.00412 (9)
Cl5	0.02426 (12)	0.01906 (12)	0.01887 (10)	0.00237 (10)	0.01270 (9)	0.00438 (9)
Cl1	0.02039 (11)	0.01434 (10)	0.02008 (11)	0.00533 (9)	0.00767 (9)	0.00351 (9)
Cl3	0.02241 (13)	0.03108 (15)	0.02344 (13)	−0.01235 (12)	0.00946 (11)	−0.00656 (11)
Cl4	0.02828 (13)	0.01860 (12)	0.02112 (11)	0.00725 (10)	0.01163 (10)	0.00335 (9)
N1	0.0145 (4)	0.0146 (4)	0.0109 (3)	0.0009 (3)	0.0052 (3)	0.0020 (3)
N2	0.0138 (3)	0.0133 (4)	0.0110 (3)	0.0011 (3)	0.0054 (3)	−0.0002 (3)
C8	0.0124 (4)	0.0121 (4)	0.0103 (3)	0.0013 (3)	0.0047 (3)	0.0003 (3)
C9	0.0200 (4)	0.0178 (5)	0.0126 (4)	−0.0006 (4)	0.0054 (3)	−0.0040 (3)
C7	0.0249 (5)	0.0150 (5)	0.0138 (4)	0.0053 (4)	0.0099 (4)	0.0039 (3)
C3	0.0131 (4)	0.0120 (4)	0.0101 (3)	−0.0003 (3)	0.0050 (3)	0.0010 (3)
C6	0.0229 (5)	0.0152 (4)	0.0172 (4)	0.0058 (4)	0.0092 (4)	0.0032 (4)
C1	0.0212 (5)	0.0290 (6)	0.0122 (4)	−0.0021 (4)	0.0015 (4)	0.0043 (4)
C5	0.0241 (5)	0.0123 (4)	0.0190 (5)	0.0013 (4)	0.0112 (4)	−0.0005 (3)
C10	0.0150 (4)	0.0256 (5)	0.0216 (5)	−0.0018 (4)	0.0101 (4)	−0.0058 (4)
C4	0.0217 (5)	0.0143 (4)	0.0151 (4)	0.0019 (4)	0.0109 (4)	−0.0003 (3)
C2	0.0190 (4)	0.0194 (5)	0.0216 (5)	−0.0018 (4)	0.0106 (4)	0.0053 (4)

### (R,R)-N1,N1,N2,N2-Tetramethylcyclohexane-1,2-bis(aminium) tetrachloridoferrate chloride (1a) Geometric parameters (Å, º)

**Table:**

Fe1—Cl2	2.1989 (3)	C7—C6	1.5290 (15)
Fe1—Cl5	2.1913 (3)	C3—H3	1.0000
Fe1—Cl3	2.1891 (3)	C3—C4	1.5310 (14)
Fe1—Cl4	2.2191 (3)	C6—H6A	0.9900
N1—C3	1.5237 (13)	C6—H6B	0.9900
N1—C1	1.5004 (14)	C6—C5	1.5224 (16)
N1—C2	1.4939 (13)	C1—H1A	0.9800
N1—H1	0.891 (19)	C1—H1B	0.9800
N2—C8	1.5245 (13)	C1—H1C	0.9800
N2—C9	1.4978 (13)	C5—H5A	0.9900
N2—C10	1.4985 (14)	C5—H5B	0.9900
N2—H2	0.900 (18)	C5—C4	1.5269 (15)
C8—H8	1.0000	C10—H10A	0.9800
C8—C7	1.5358 (14)	C10—H10B	0.9800
C8—C3	1.5458 (13)	C10—H10C	0.9800
C9—H9A	0.9800	C4—H4A	0.9900
C9—H9B	0.9800	C4—H4B	0.9900
C9—H9C	0.9800	C2—H2A	0.9800
C7—H7A	0.9900	C2—H2B	0.9800
C7—H7B	0.9900	C2—H2C	0.9800
			
Cl2—Fe1—Cl4	108.335 (12)	C8—C3—H3	107.6
Cl5—Fe1—Cl2	107.664 (13)	C4—C3—C8	110.77 (8)
Cl5—Fe1—Cl4	108.833 (12)	C4—C3—H3	107.6
Cl3—Fe1—Cl2	111.878 (13)	C7—C6—H6A	109.8
Cl3—Fe1—Cl5	112.196 (13)	C7—C6—H6B	109.8
Cl3—Fe1—Cl4	107.837 (15)	H6A—C6—H6B	108.2
C3—N1—H1	110.2 (11)	C5—C6—C7	109.49 (9)
C1—N1—C3	111.89 (9)	C5—C6—H6A	109.8
C1—N1—H1	104.8 (11)	C5—C6—H6B	109.8
C2—N1—C3	115.23 (8)	N1—C1—H1A	109.5
C2—N1—C1	109.82 (9)	N1—C1—H1B	109.5
C2—N1—H1	104.1 (11)	N1—C1—H1C	109.5
C8—N2—H2	109.8 (10)	H1A—C1—H1B	109.5
C9—N2—C8	112.18 (8)	H1A—C1—H1C	109.5
C9—N2—C10	109.41 (8)	H1B—C1—H1C	109.5
C9—N2—H2	101.9 (11)	C6—C5—H5A	109.8
C10—N2—C8	116.03 (8)	C6—C5—H5B	109.8
C10—N2—H2	106.4 (10)	C6—C5—C4	109.44 (9)
N2—C8—H8	108.0	H5A—C5—H5B	108.2
N2—C8—C7	109.45 (8)	C4—C5—H5A	109.8
N2—C8—C3	112.87 (8)	C4—C5—H5B	109.8
C7—C8—H8	108.0	N2—C10—H10A	109.5
C7—C8—C3	110.43 (8)	N2—C10—H10B	109.5
C3—C8—H8	108.0	N2—C10—H10C	109.5
N2—C9—H9A	109.5	H10A—C10—H10B	109.5
N2—C9—H9B	109.5	H10A—C10—H10C	109.5
N2—C9—H9C	109.5	H10B—C10—H10C	109.5
H9A—C9—H9B	109.5	C3—C4—H4A	109.4
H9A—C9—H9C	109.5	C3—C4—H4B	109.4
H9B—C9—H9C	109.5	C5—C4—C3	111.00 (8)
C8—C7—H7A	109.1	C5—C4—H4A	109.4
C8—C7—H7B	109.1	C5—C4—H4B	109.4
H7A—C7—H7B	107.8	H4A—C4—H4B	108.0
C6—C7—C8	112.48 (9)	N1—C2—H2A	109.5
C6—C7—H7A	109.1	N1—C2—H2B	109.5
C6—C7—H7B	109.1	N1—C2—H2C	109.5
N1—C3—C8	113.18 (8)	H2A—C2—H2B	109.5
N1—C3—H3	107.6	H2A—C2—H2C	109.5
N1—C3—C4	109.98 (8)	H2B—C2—H2C	109.5
			
N1—C3—C4—C5	177.07 (8)	C7—C6—C5—C4	−59.87 (12)
N2—C8—C7—C6	−178.78 (9)	C3—C8—C7—C6	−53.93 (12)
N2—C8—C3—N1	−60.24 (11)	C6—C5—C4—C3	60.65 (12)
N2—C8—C3—C4	175.71 (8)	C1—N1—C3—C8	158.64 (9)
C8—C7—C6—C5	57.65 (13)	C1—N1—C3—C4	−76.88 (11)
C8—C3—C4—C5	−57.08 (11)	C10—N2—C8—C7	59.79 (11)
C9—N2—C8—C7	−66.97 (11)	C10—N2—C8—C3	−63.63 (11)
C9—N2—C8—C3	169.61 (9)	C2—N1—C3—C8	−74.97 (11)
C7—C8—C3—N1	176.89 (8)	C2—N1—C3—C4	49.51 (11)
C7—C8—C3—C4	52.84 (11)		

### (R,R)-N1,N1,N2,N2-Tetramethylcyclohexane-1,2-bis(aminium) tetrachloridoferrate chloride (1a) Hydrogen-bond geometry (Å, º)

**Table:**

*D*—H···*A*	*D*—H	H···*A*	*D*···*A*	*D*—H···*A*
N1—H1···Cl1	0.891 (19)	2.133 (19)	3.0189 (11)	172.7 (19)
N2—H2···Cl1	0.900 (18)	2.135 (17)	3.0208 (10)	165 (14)
